# Apigenin Induces Apoptosis and Inhibits Migration in Human Cholangiocarcinoma Cells

**DOI:** 10.3390/toxics13020112

**Published:** 2025-01-30

**Authors:** Mayurachat Kaewmanee, Temduang Limpaiboon, Nipaporn Ngernyuang

**Affiliations:** 1 Chulabhorn International College of Medicine, Thammasat University, Pathumthani 12120, Thailand; may_mtmu@yahoo.com; 2 Centre for Research and Development of Medical Diagnostic Laboratories, Faculty of Associated Medical Science, Khon Kaen University, Khon Kaen 40002, Thailand; temduang@kku.ac.th; 3 Thammasat University Research Unit in Biomedical Science, Thammasat University, Pathumthani 12120, Thailand

**Keywords:** apigenin, anticancer, cholangiocarcinoma, apoptosis, anti-migration

## Abstract

Cholangiocarcinoma (CCA) is a rare and highly aggressive cancer of the biliary tract, associated with poor clinical outcomes due to late diagnosis, extensive metastasis, drug resistance, and limited treatment options. Apigenin, a natural flavonoid, has been found to exhibit anticancer properties in several types of human cancer cells. Therefore, apigenin may be relevant to developing chemotherapeutic agents for cancer treatment. In this study, we examined the effects of apigenin on cell viability, cell cycle distribution, apoptosis, and cell migration in human CCA cell lines (KKU-M055) under *in vitro* conditions. The results demonstrate that apigenin significantly suppressed specific CCA cell proliferation by inducing cell cycle arrest at the G2/M phase and promoting cell apoptosis in KKU-M055 cells while exhibiting low toxicity in immortalized MMNK1 cells. Apigenin enhanced apoptotic features, including nuclear fragmentation and the loss of mitochondrial membrane potential. Furthermore, apigenin induced the apoptosis of KKU-M055 cells in both extrinsic and intrinsic pathways by activating caspase-8, -9, and -3/7. Moreover, apigenin inhibited KKU-M055 migration. Our study suggests apigenin as a promising candidate for treating CCA, and these findings provide theoretical support for the further development and potential application of apigenin in clinical CCA therapy.

## 1. Introduction

Cholangiocarcinoma (CCA) is a hepatobiliary cancer that is extremely aggressive and originates from the epithelial cells of the bile ducts. Although CCA is rare worldwide, one report indicated high incidence rates in East and Southeast Asia, particularly in northeast Thailand [1]. In Thailand, the primary risk factor of CCA is infection with the liver fluke *Opisthorchis viverrini* (OV), which is a carcinogen that causes the normal bile duct to transform into a tumor [2]. It has been difficult to detect and diagnose CCA early since the disease frequently manifests at an advanced or locally advanced stage. The only potentially curative treatment for CCA patients is surgical resection, but most patients are not eligible for this procedure because their condition is advanced at diagnosis [3]. In recent years, treatment strategies for advanced CCA have been explored, including chemotherapy, radiotherapy, and combination chemoradiotherapy. The standard recommended treatment for unresectable or advanced CCA consists of a combination of gemcitabine and cisplatin (GC). This regimen has been shown to improve both overall survival (OS) and progression-free survival (PFS) compared to gemcitabine or cisplatin alone [4]. However, this treatment often causes side effects due to damage to the surrounding healthy tissues or organs. Moreover, the long-term prognosis of CCA patients treated with systemic GC treatment remains poor due to drug resistance [5]. Therefore, developing innovative treatment drugs is important for improving the survival rates of CCA patients. For over half a century, bioactive phytochemical compounds have been essential in cancer chemotherapy and chemoprevention [6]. Apigenin is a naturally occurring flavonoid compound (4′,5,7-trihydroxyflavone, C_15_H_10_O_5_) found in fruits and vegetables. Apigenin has been demonstrated to possess various pharmacological effects, such as anti-inflammatory [7], anti-viral [8,9], and anti-oxidative effects [10,11]. Apigenin has been shown in several studies to have broad anticancer effects through various mechanisms, including inhibiting proliferation, apoptosis induction, autophagy induction, modulating the signaling pathway involved in cancer progression, and angiogenesis suppression [12,13,14,15,16,17,18,19,20]. These effects have been observed in various types of cancers, including colorectal cancer [12], breast cancer [13], liver cancer [14,15], lung cancer [16], cervical cancer [17], ovarian cancer [18], prostate cancer [19], and skin cancer [20]. Accordingly, the biological effects of apigenin on the cell viability, apoptosis, and migration of human CCA cell line KKU-M055 were investigated in the present study. This research aimed to assess the potential of apigenin as a novel therapeutic agent for treating CCA.

## 2. Materials and Methods

### 2.1. Cell Culture

All cell lines, including the KKU-M055 cell line, a human intrahepatic CCA (moderately differentiated adenocarcinoma) (CVCL_M258), and an immortalized human cholangiocyte cell line MMNK1 (CVCL_M266), were obtained from the Japanese Collection of Research Bioresources (JCRB, Osaka, Japan). Cell lines were grown in a Dulbecco’s Modified Eagle’s Medium high-glucose (DMEM-HG) medium supplemented with 1% *v*/*v* penicillin/streptomycin and 10% *v*/*v* fetal bovine serum (FBS) (all from Life Technologies, Paisley, UK). All cells were cultured and maintained in a humidified incubator at 37 °C with 5% CO_2_.

### 2.2. Cell Viability Assay

Cell viability was assessed to evaluate cytotoxicity using the CellTiter 96^®^ AQueous One Solution Cell Proliferation Assay (MTS assay), which utilizes 3-(4,5-dimethylthiazol-2-yl)-5-(3-carboxymethoxyphenyl)-2-(4-sulfophenyl)-2H-tetrazolium (Promega, Madison, WI, USA). Briefly, KKU-M055 cells were seeded in 96-well plates at 5 × 10^3^ cells/well, then incubated at 37 °C with 5% CO_2_ and allowed to form a confluent monolayer for 24 h. The medium was replaced with a fresh medium that included triplicates of apigenin (C_15_H_10_O_5_, Sigma-Aldrich, St. Louis, MO, USA) at varying concentrations (20, 40, 60, 80, 100, and 120 μM), while untreated cells served as the control. The cells were incubated at 37 °C with 5% CO_2_, and after 24 and 48 h, an MTS assay was conducted in each well. The cells were incubated at 37 °C with 5% CO_2_ for 1 h. The absorbance of samples was measured at 490 nm using a microplate reader (Thermo Varioskan Flash Multi Detection microplate reader, Thermo Fisher Scientific, Waltham, MA, USA). The cell viability percentage was calculated relative to untreated controls, and the half-maximal inhibitory concentration (IC50) was determined using a linear regression equation based on the viability data.

### 2.3. Cell Cycle Distribution Assay

For cell cycle analysis, KKU-M055 cells were treated with 80 µM apigenin (the IC50 concentration) for 24 h in triplicate, while untreated cells served as the control. A BD Cycletest™ Plus Reagent Kit was used following the manufacturer’s instructions (BD Biosciences, San Jose, CA, USA). Cell cycle distribution was analyzed using a BD FACSAria II flow cytometer 9.0 (BD Biosciences), and data were processed using BD FACSDiva software 8.0.2 (BD Biosciences).

### 2.4. Apoptosis Assay

The induction of apoptosis in KKU-M055 cells by apigenin was evaluated using flow cytometry. In brief, 2.5 × 10^5^ cells in a 25 cm^2^ culture flask were treated with 80 μM apigenin for 24 h in triplicate, with untreated cells serving as the control. After treatment, the cells were collected and stained using a FITC Annexin V Apoptosis Detection Kit I (BD Biosciences, San Jose, CA, USA) according to the manufacturer’s instructions. The stained cells were then analyzed using a BD FACSAria II flow cytometer 9.0 (BD Biosciences), and data were processed using BD FACSDiva software 8.0.2 (BD Biosciences).

### 2.5. Apoptotic Cell Staining

Nuclear morphology was examined to evaluate apoptosis using the fluorescent DNA-binding dye Hoechst 33258 solution (5 μg/mL) (Sigma-Aldrich). In brief, 5 × 10^4^ cells in a 24-well plate were treated with 80 μM of apigenin for 24 h in triplicate, while untreated cells served as the control. After incubation, the cells were stained with Hoechst 33258 solution at room temperature for 10 min. The stained cells were then observed under a fluorescence microscope to examine the morphology of apoptotic nuclei.

To assess mitochondrial function, a mitochondrial transmembrane potential assay was performed using Rhodamine 123 (Rho 123 staining) (Sigma-Aldrich). In brief, Hoechst 33258-stained cells were incubated with Rho 123 (2.5 μg/mL) at room temperature in the dark for 15 min. After the incubation period, the cells were washed with PBS to remove excess dye. The mitochondrial membrane potential was then evaluated by observing under a fluorescence microscope.

### 2.6. Caspase Activity Assay

The activity of caspase-8, -9, and -3/7 was detected using the Caspase-Glo^®^ caspase-8, -9, and -3/7 assay kits (Promega, Madison, WI, USA). Briefly, 5 × 10^3^ cells in a black wall, clear bottom 96-well plate were treated with 80 μM apigenin in triplicate, with untreated cells serving as the control. After 24 h of treatment, Caspase-Glo reagent was added to each well and incubated at room temperature for 30 min. Luminescence was subsequently measured using a microplate reader (Thermo Varioskan Flash Multi Detection microplate reader, Thermo Fisher Scientific). Data are expressed as the percentage change compared to the untreated control, which was set to 100% activity.

### 2.7. Cell Migration Assay

The effects of apigenin on cell migration were assessed using a Transwell system (Costar Corning, Kennebunk, ME, USA). In brief, cell suspensions with a density of 2 × 10^5^ were prepared in a serum-free medium and seeded into the upper chamber of the Transwell in triplicate. The lower chamber was filled with 10% FBS medium, and 80 μM apigenin was added as a chemoattractant in triplicate, with untreated cells serving as the control group. Following incubation, non-migrating cells on the upper side of the filter were carefully removed using a cotton swab. Migrating cells that had adhered to the underside of the filter were fixed with methanol for 10 min and stained with 2.5% crystal violet for 30 min at room temperature. The number of migrating cells was counted in nine randomly selected and non-overlapping fields under a light microscope at 200× magnification.

### 2.8. Statistical Analysis

Data were analyzed using SPSS version 20 software (IBM Corp., Armonk, NY, USA). The data were expressed as the mean ± standard deviation (SD). Group comparisons were analyzed using a two-tailed Student’s *t*-test with *p* < 0.05 considered statistically significant.

## 3. Results

### 3.1. Apigenin Inhibited Cell Proliferation of CCA Cells

**Figure 1:**
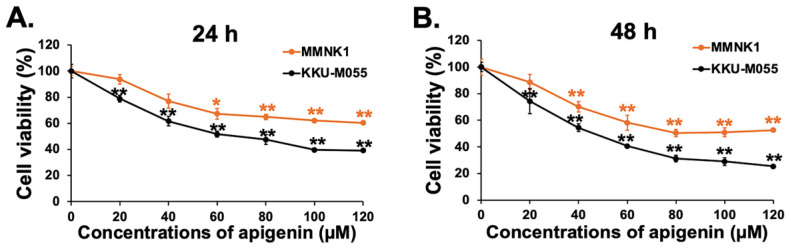
The cytotoxicity effects of apigenin on MMNK1 immortalized human cholangiocyte and KKU-M055 human cholangiocarcinoma cells. The percentage of cell viability after apigenin treatments of 24 h (**A**) and 48 h (**B**) was calculated relative to the untreated control group. Data represent the mean ± SD of three independent observations. *p* < 0.05 was considered statistically significant (* *p* < 0.05; ** *p* < 0.01).

Cell viability in vitro was assessed using an MTS assay to show the inhibitory effects of apigenin on cell proliferation. MMNK1, an immortalized human cholangiocyte cell line, and KKU-M055, a human CCA cell line, were treated with different concentrations of apigenin, ranging from 20 to 120 μM for 24 and 48 h. The results demonstrated that apigenin effectively inhibited the proliferation of KKU-M055 cells in a dose- and time-dependent manner (Figure 1). After 24 and 48 h of treatment, the IC50 values of apigenin for KKU-M055 cells were 78 μM and 61 μM, respectively. These values were approximately 1.7-fold lower than those for MMNK1 cells (132 μM and 100 μM, respectively), suggesting that apigenin is less toxic to immortalized cholangiocyte cells. Based on the MTS results, a concentration of 80 μM apigenin, corresponding to its IC50 value, was selected for further studies.

### 3.2. Apigenin Causes Cell Cycle Arrest in CCA Cells

**Figure 2:**
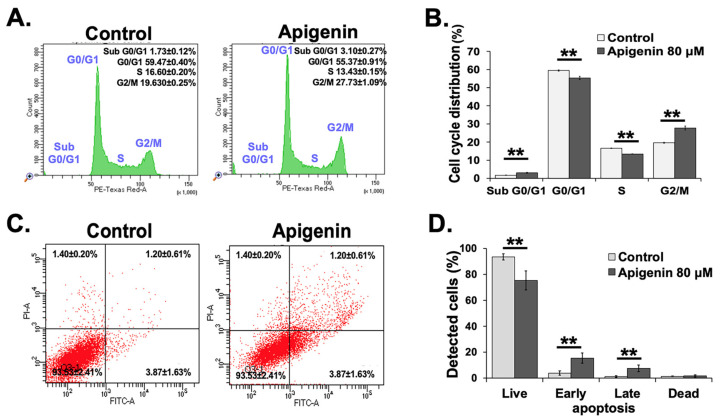
Effects of apigenin on cell cycle progression and apoptosis in KKU-M055 cells using flow cytometry. KKU-M055 cells were either untreated or treated with apigenin at IC50 concentrations for 24 h. (**A**) Representative histograms illustrating distribution of cell cycle phases. (**B**) Quantitative data representing percentage of cells in various cell cycle phases. (**C**) Apoptosis induction was examined using flow cytometry-based Annexin V and PI staining. (**D**) Quantitative data on apoptosis percentages in untreated and apigenin-treated cells. Data are shown as means ± SD from three independent experiments. ** *p* < 0.01 indicates statistically significant differences.

The reduction in cell viability may result from the induction of cell growth arrest and/or apoptosis. This study aimed to determine the effects of apigenin on cell cycle progression and apoptosis in CCA cells. KKU-M055 cells were treated with 80 µM apigenin for 24 h to evaluate whether apigenin induces cell cycle arrest. Flow cytometric analysis revealed a significant increase in the number of cells in the G2/M phase (from 19.63 ± 0.25 to 27.73 ± 1.09%). In contrast, a corresponding decrease in the proportion of cells was observed in the G0/G1 phase (from 59.47 ± 0.40% to 55.37 ± 0.91%) and S phase (from 16.60 ± 0.20% to 13.43 ± 0.15%) compared to untreated cells (*p* < 0.01), suggesting that apigenin induces a G2/M cell cycle arrest (Figure 2A,B).

### 3.3. Promotion of Apoptosis by Apigenin Through Activation of Extrinsic and Intrinsic Pathways in KKU-M055 Cells

To examine whether apigenin induced apoptotic cell death in KKU-M055 cells, a flow cytometric analysis of phosphatidylserine (PS) externalization was used to detect cell apoptosis. PS, a type of glycerophospholipid, is a major component of the plasma membrane. In living cells, PS is typically restricted to the inner leaflet of the plasma membrane. PS exposure on the outer plasma membrane is a hallmark of apoptosis, a programmed cell death process distinguished by cell shrinkage, chromatin condensation, the fragmentation of nuclear DNA, and preserved plasma membrane integrity [21]. When cells undergo an early stage of apoptosis, the event that occurs is PS externalization on the cell surface, detected by Annexin V staining. After the progression to the late stages of apoptosis, PI has labeled apoptotic cells due to the membrane integrity of the cells being lost [22]. Figure 2C,D demonstrate that in the untreated group, 93.53 ± 2.41% of cells remained viable, with less than 7% exhibiting apoptosis. In contrast, treatment with an IC50 concentration of apigenin for 24 h resulted in a significant shift, with 75.33 ± 7.29% of cells remaining viable, while 24.67 ± 7.43% of cells displayed apoptotic characteristics. This suggests that apigenin induced a notable increase in apoptosis in the KKU-M055 cells.

**Figure 3:**
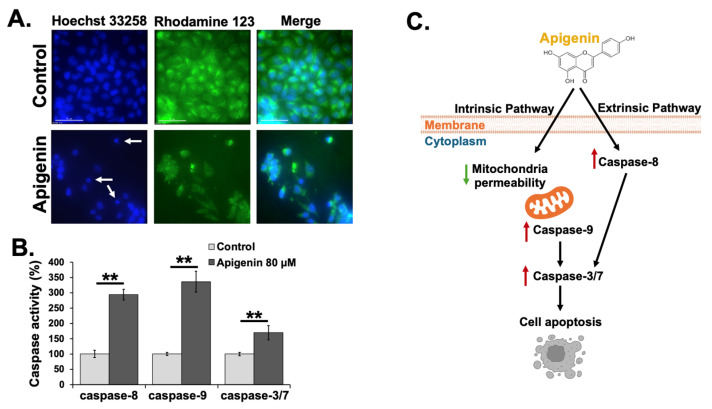
Apigenin-induced apoptosis characteristics and caspase activity in KKU-M055 cells. (**A**) Fluorescence micrographs showing Hoechst 33258 (first column) and Rho 123 (middle column) staining in untreated and apigenin-treated KKU-M055 cells. Morphological changes, including nuclear condensation and fragmentation, are indicated by white arrows. (**B**) Caspase activity induced by apigenin treatment, with activities of caspase-8, -9, and -3/7 assessed using luminescence assay. Values are expressed as mean ± SD from three independent experiments. ** *p* < 0.01 are considered statistically significant. (**C**) Proposed mechanisms of apigenin-induced apoptosis in KKU-M055 cells, showing caspase cascade activation via both extrinsic and intrinsic pathways. The red arrows indicate an increase relative to the control, while the green arrows indicate a decrease relative to the control.

The growth inhibitory effects of apigenin on cancer cells were mediated through apoptosis, as demonstrated by hallmark features such as nuclear condensation, the formation of apoptotic bodies, and DNA fragmentation. To further confirm these apoptotic characteristics, cells were stained with Hoechst 33258, enabling the visualization of nuclear morphological changes associated with apoptosis under a fluorescence microscope. Apigenin triggered an apoptotic response in KKU-M055 cells compared to untreated controls. As shown in Figure 3A (first column), apigenin-treated KKU-M055 cells exhibited nuclear condensation and fragmentation (white arrow), which are the characteristic features of apoptosis, indicating that apigenin effectively induces apoptosis in CCA cells.

To determine whether the observed apoptosis is linked to the loss of mitochondrial membrane potential (ΔΨm), cells were stained with Rho 123 dye, a marker of the earliest events in the apoptotic cascade. Apigenin treatment resulted in a reduction in Rho 123 fluorescence compared to untreated cells (Figure 3A, middle column), suggesting that apigenin triggered mitochondrial damage-associated apoptosis in KKU-M055 cells.

### 3.4. Effects of Apigenin on Caspase Activity in KKU-M055 Cells

To assess the impact of apigenin on the activities of key caspases (caspase-8, -9, and -3/7) in CCA cells, KKU-M055 cells were treated with an IC50 concentration of apigenin for 24 h, and caspase activities were measured using the Caspase-Glo kits. As shown in Figure 3B, the treatment with apigenin resulted in a significant increase in the activities of caspase-8, -9, and -3/7 compared to the untreated cells, with the differences being statistically significant (*p* < 0.01). These results suggested that apigenin induces apoptosis in CCA cells through both extrinsic and intrinsic apoptotic pathways by enhancing the activity of caspases-8, -9, and -3/7 (Figure 3C).

### 3.5. Inhibition of KKU-M055 Cell Migration by Apigenin

**Figure 4:**
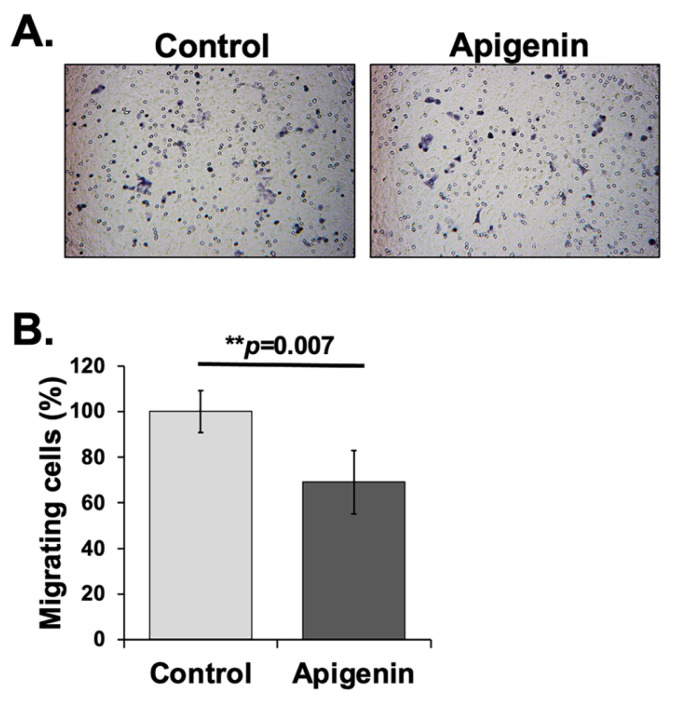
Apigenin inhibits KKU-M055 migration. (**A**) Representative image showing effects of apigenin on cell migration, as assessed using Transwell migration assay. (**B**) Quantitative data showed significant reduction in number of migrating cells treated with apigenin compared to untreated control. Data are represented as mean ± SD. ** *p* < 0.01 indicates statistically significant compared to control.

To assess whether apigenin can inhibit the migration of CCA cells, its effect on cell motility was evaluated using a Transwell migration assay. As shown in Figure 4, apigenin treatment significantly reduced the migration of KKU-M055 cells by 38.11 ± 13.86% compared to untreated cells. These results suggest that apigenin effectively inhibits the migration of CCA cells.

## 4. Discussion

CCA is a rare and aggressive cancer with a poor prognosis, and its incidence is rising. This highlights the critical need for the development of novel therapeutic compounds to improve outcomes and address the current lack of effective treatment options [23]. Apigenin, a natural dietary flavonoid, is found in various vegetables, herbs, and fruits. It has been demonstrated to suppress tumor growth by inhibiting cell proliferation [12,13,14,15,16,17,18,19,20]. In this study, we demonstrated the chemotherapeutic potential of apigenin against CCA in vitro. The human CCA cell line KKU-M055 derived from a human intrahepatic CCA was selected as a model for our investigation. Our findings revealed that apigenin inhibited the proliferation of KKU-M055 cells while exhibiting low toxicity in immortalized human cholangiocyte cell line MMNK1 cells. Additionally, we tested cell viability on another CCA cell line (KKU-M213A) (CVCL_M261, JCRB), and the results were similar to those observed with KKU-M055, indicating that apigenin has a comparable effect on KKU-M213A (Figure S1). These findings suggest that apigenin possesses chemotherapeutic potential in the treatment of CCA. Consistent with our findings, previous studies found that apigenin suppresses cell proliferation and causes apoptosis in various cancer cell types [12,13,14,15,16,17,18,19,20], while apigenin displays low cytotoxicity in normal cells [24,25].

Cell cycle dysregulation is a hallmark of tumorigenesis, caused by the breakdown of multiple tightly regulated checkpoints. The G2/M checkpoint prevents cells from entering mitosis when DNA damage is present, allowing for time for repair and preventing the proliferation of damaged cells [26]. Apoptotic pathways are triggered to eliminate affected cells if the damage is irreparable [27]. Our findings reveal that apigenin suppressed the growth of human CCA cells by inducing the G2/M-phase arrest of the cell cycle. Several studies have demonstrated that apigenin inhibits cancer cell proliferation by modulating the cell cycle and inducing a G2/M phase in various cancer cell types. These include hepatocellular carcinoma [14,15], human colorectal carcinoma HCT116 cells [28], human breast cancer cell line MDA-MB-231 cells [29], renal cell carcinoma ACHN cells [30], adenoid cystic carcinoma ACC-2 cells [31], and human papillary thyroid carcinoma BCPAP cells [32]. Furthermore, apigenin has been reported to induce cell cycle arrest at the G0/G1 or S phase checkpoints in LNCaP human prostate cancer cells [24]. In the SCC-25 oral squamous cell carcinoma cell line, apigenin treatment was found to cause cell cycle arrest at both the G0/G1 and G2/M phase checkpoints [33]. These findings suggest that the effects of apigenin on cell cycle progression may vary depending on the specific characteristics of the cell line.

Apoptosis is a form of programmed cell death (PCD) that plays a crucial role in physiological development and is essential for maintaining tissue homeostasis [34]. The activation of caspases is the hallmark of the investigation of apoptosis pathways. Caspase-8 activation is associated with the extrinsic pathway, while caspase-9 activation mediates the intrinsic pathway. In the extrinsic pathway, cleaved caspase-8 activates caspase-3, thereby initiating apoptosis. In the intrinsic pathway, caspase-9 triggers the release of cytochrome c from mitochondria in response to various agents or insults, which subsequently activates caspase-3, a common downstream effector in both pathways [35]. In our study, we demonstrated that apigenin treatment significantly increased the apoptosis rates of KKU-M055 cells compared to the control group. Furthermore, apigenin treatment enhanced the expression of caspase-8, -9, and -3/7, which are key markers of apoptotic activity. Our findings are consistent with previous reports, which have demonstrated that apigenin induced cancer cell apoptosis through both extrinsic and intrinsic pathways [36,37]. Zhao et al. found that apigenin promotes the apoptosis pathway by activating cleaved caspase-3 and cleaved PARP proteins [20]. Additionally, Seo et al. observed that apigenin induces apoptosis through the extrinsic pathway by activating p53 and suppressing STAT3 and NF-κB signaling in human epidermal growth factor receptor 2 (HER2)-overexpressing breast cancer cells [38]. Furthermore, we observed that apigenin induced DNA fragmentation and the loss of mitochondrial membrane potential (ΔΨm) in KKU-M055 cells. These observations are in line with previous research demonstrating that apigenin can induce similar effects in other cancer cell types, including human breast cancer MDA-MB-453 cells [39] and human bladder cancer T-24 cells [40]. This consistency suggests that apigenin has an impact on DNA integrity, and mitochondrial function may be a common mechanism across different cancer cell lines.

Cancer cell migration and invasion play an important role in tumor metastasis, which is an adverse prognostic factor contributing to cancer-related mortality [41]. We found that apigenin significantly inhibited KKU-M055 cell migration. Consistent with other reports, apigenin has been demonstrated to be an effective agent for inhibiting cell migration in CD44+ prostate cancer cells [42] and colorectal cancer cells (DLD1 and SW480) [43]. Moreover, Qin et al. found that apigenin inhibited the invasion and migration of human hepatocellular carcinoma cells (Bel-7402 and PLC) by suppressing the NF-κB/Snail pathway and reversing the upregulation of epithelial–mesenchymal transition (EMT) marker levels [44].

## 5. Conclusions

In conclusion, our study demonstrates that apigenin significantly suppressed KKU-M055 CCA cell proliferation in a dose- and time-dependent manner by inducing the G2/M-phase arrest of the cell cycle. Additionally, apigenin was shown to activate both the extrinsic and intrinsic pathways of apoptosis via the activation of caspase-8, -9, and -3/7. Moreover, apigenin also inhibits cancer cell migration. All these results indicate that apigenin can be used as a chemopreventive agent in CCA due to its characteristics of safety, low toxicity, and high efficiency. To the best of our knowledge, this is the first report showing the anticancer and anti-migration effects of apigenin in CCA cancer in vitro. However, it is important to note that the analysis was conducted using a single cancer cell line. Future studies involving multiple types of CCA cell lines to investigate the mechanisms behind apigenin-induced cell inhibition, as well as evaluating its effects on in vivo models, may provide a more comprehensive understanding of its therapeutic potential and outcomes specific to CCA.

## Data Availability

The original contributions presented in this study are included in the article/Supplementary Material. Further inquiries can be directed to the corresponding author.

## Supplementary Materials

The following supporting information can be downloaded at https://www.mdpi.com/article/10.3390/toxics13020112/s1, Figure S1: Cytotoxicity effect of apigenin on MMNK1 immortalized human cholangiocyte, KKU-M055, and KKU-M213A human cholangiocarcinoma cells.
