# Supplementary material for: Apigenin Induces Apoptosis and Inhibits Migration in Human Cholangiocarcinoma Cells
Source: Toxics. 2025 Jan 30;13(2):112. doi: 10.3390/toxics13020112 (PMC11860412; doi:10.3390/toxics13020112)
Supplement: Supplementary file 1 [file toxics-13-00112-s001.zip › toxics-3430042-supplementary.pdf]

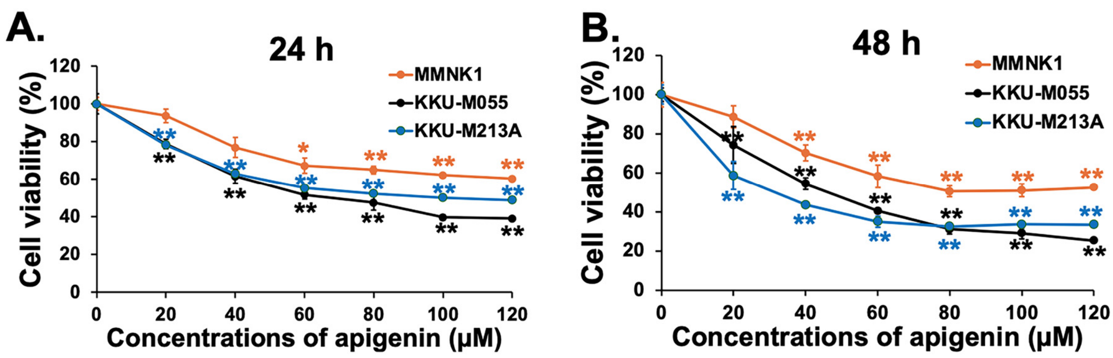

Figure S1. Cytotoxicity effect of apigenin on MMNK1 immortalized human cholangiocyte, KKU-M055, and KKU-M213A human cholangiocarcinoma cells. The percentage of cell viability after apigenin treatment 24 h (A.) and 48 h (B.) was calculated relative to the untreated control group. Data represents the mean±SD of three independent observations. A p<0.05 of less than 0.05 was considered statistically significant (*$p<0.05$; **$p<0.01$).
